# An Innovative Model of ISS‐Based Multiple Fractures and Gastrointestinal Dysfunction Related to c‐Kit Protein Expression on Interstitial Cells of Cajal

**DOI:** 10.1111/os.13599

**Published:** 2023-03-15

**Authors:** Shi‐Jie Meng, Meng‐Qiang Fan, Jian‐Sheng Qian, Jing‐Wen Zhang, Hui‐Hui Xu, Yang Zheng, Wei‐Qiang Zhao, Le‐Tian Shan, Jie‐Feng Huang

**Affiliations:** ^1^ The First Clinical College Zhejiang Chinese Medical University Hangzhou China; ^2^ Department of Orthopaedics & Traumatology The First Affiliated Hospital of Zhejiang Chinese Medical University Hangzhou China; ^3^ The Third Clinical College Zhejiang Chinese Medical University Hangzhou China; ^4^ Research and Development Department Cell Resource Bank and Integrated Cell Preparation Center of Xiaoshan District Hangzhou China

**Keywords:** Animal Model, c‐Kit Protein, Gastrointestinal Dysfunction, Multiple Fracture

## Abstract

**Objective:**

Gastrointestinal dysfunction seriously affects the prognosis and quality of life of patients with multiple fractures. However, experimental evidence of this relationship is lacking. Here we describe a newly developed mouse model of postoperative gastrointestinal dysfunction after multiple fractures.

**Methods:**

Trauma severity was assessed using the injury severity score (ISS). Based on the ISS, a multiple fracture model was established in mice as follows: limb fractures with pelvic fractures and multiple rib fractures; limb fractures with multiple rib fractures; closed fracture of both forelegs with pelvic fracture and rib fractures; closed limb fractures; limb fracture with pelvic fracture; spinal fractures; hind leg fractures with pelvic fractures; pelvic fracture with multiple rib fractures; closed fracture of both fore legs with pelvic fracture; and closed fracture of both fore legs with multiple rib fractures. In each model group, gastrointestinal motility was assayed and the histopathology of the small intestine was examined. Western blot and immunohistochemical analyses of jejunal tissue were performed to detect c‐kit protein expression, the level of which was compared with that of a control group. The results of ANOVA are expressed as mean ± standard deviation.

**Results:**

In mice with multiple fractures, food intake was greatly reduced, consistent with histopathological evidence of an injured intestinal epithelium. The jejunal tissue of mice in groups a, c, f, and h was characterized by extensively necrotic and exfoliated intestinal mucosal epithelium and inflammatory cell infiltration in the lamina propria. In the gastrointestinal function assay, gastrointestinal motility was significantly reduced in groups a, b, c, f, and g; these group also had a higher ISS (*p* < 0.01). The expression of c‐kit protein in groups with gastrointestinal dysfunction was significantly up‐regulated (*p* < 0.001) compared with the control group. The close correlation between c‐kit expression and the ISS indicated an influence of trauma severity on gastrointestinal motility.

**Conclusion:**

Gastrointestinal dysfunction after multiple fractures was successfully reproduced in a mouse model. In these mice, c‐kit expression correlated with gastrointestinal tissue dysfunction and might serve as a therapeutic target.

## Graphical abstract

This study innovatively established a mouse model to simulate postoperative gastrointestinal dysfunction after multiple fractures. ISS‐based gastrointestinal dysfunction model after multiple fractures was related to c‐kit protein from interstitial cells of Cajal.

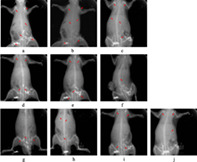

## Introduction

Fractures are the most common form of large‐organ traumatic injury and their incidence has increased in recent years.^1^ The recovery period for patients with fractures is generally long and the risk of complications is high,^2^ resulting in longer treatment cycles and a higher cost of therapy.^3^, ^4^ Perioperative complications often reflect the severity of the fracture trauma itself.^5^ Accordingly, assessment of trauma severity can provide information on the incidence of complications. The injury severity score (ISS), introduced in 1974,^6^ divides the human body into six parts: head and neck, face, chest, abdomen, limbs/pelvis, and body surface. Scores of <16, ≥16, and ≥25 are considered indicative of minor, serious, and critical injury, respectively.^6^, ^7^

During the perioperative period, fracture patients often develop severe abdominal distension, constipation, nausea, vomiting, and other symptoms of disturbed gastrointestinal motility, which lead to a poor prognosis and low quality of life.^8^, ^9^, ^10^, ^11^, ^12^ The pathogenesis of the gastrointestinal disturbances in fracture patients is thought to be mediated by the inhibitory sympathetic reflex system, which is activated by trauma; it increases the activity of the sympathetic nervous system in the gastrointestinal tract and inhibits gastrointestinal motility by suppressing excitatory neurons in the gastrointestinal plexus.^13^ An increase in the blood glucose level, as a stress response to trauma, also inhibits gastrointestinal motility.^14^

The interstitial cells of Cajal (ICC) act as pacemaker cells and are widely distributed in the gastrointestinal tract. Their functions include signal generation, the conductance of slow‐wave potentials, and neural regulation.^15^ Gastrointestinal motility disorders are associated with a decrease in the number of ICCs and changes in their morphology and network structure.^16^ Due to their fundamental role in the gastrointestinal tract, ICCs may be an important target for gastrointestinal motility therapies. For example, electroacupuncture was recently shown to effectively alleviate ICC damage and reduce recombinant connexin 43 levels, thus improving gastrointestinal motility disorders.^17^ Gastrointestinal ICCs express the surface receptor c‐kit, which is widely used to specifically detect ICCs, thereby serving as an indicator of the degree of gastrointestinal motility.^18^, ^19^ Thus, in this study we investigated the potential relationship between the severity of trauma and expression of c‐kit protein in ICCs.

Specifically, we developed a mouse model that was used to investigate the following: the development of gastrointestinal dysfunction following multiple fractures; the link between the ISS score and severity of multiple fractures; and the potential mechanism relating multiple fractures to gastrointestinal dysfunction (assessed based on ICCs and c‐kit protein). The validity of the mouse model of gastrointestinal dysfunction after multiple fractures was demonstrated in tissue, pathological, and molecular experiments aimed at detecting the expression of gastrointestinal‐function‐related proteins. Our innovative animal model provides insight into the mechanisms underlying disturbed gastrointestinal motility in patients with multiple fractures.

## Materials and Methods

### Animal Model

Male C57BL/6 mice were obtained from Zhejiang Chinese Medical University Animal Experimental Center. To avoid the effects of stress induced by the gastrointestinal treatments, the mice were acclimated to the gavage needles twice a day, starting 3 days before the experiment.

Multiple fractures of the long bones were created in the mice as follows. The mice were anesthetized with 2% pelltobarbitalum natricum (0.3 ml/10 g per mouse, administered intraperitoneally). Surgical vascular clamps were then used to clamp both ends of the long bones of the hind and forelegs. Closed fractures were created and confirmed immediately by X‐ray. Pelvic, rib, and spinal fractures were created using vascular forceps to exert an external force on the lumbar vertebral body, ribs, and pelvis. The mice were kept warm using an electric heater until they were fully awake. They were then transferred to separate cages.

The mice were grouped according to the fracture combination and ISS (Table 1). To eliminate the influence of dead mice on the experimental results, each model group consisted of 15 mice. The control group contained 10 mice. After the surgical procedure, liquid food was provided continuously for 3 days.

**TABLE 1:** Injury severity score (ISS) data for the 10 multiple fracture groups

Group	Multiple fracture types	ISS
a	Limb fracture with pelvic fracture and multiple rib fractures	34
b	Limb fracture with multiple rib fractures	25
c	Closed fracture of both fore legs with pelvic fracture and rib fractures	34
d	Closed fracture of limbs	16
e	Limb fracture with pelvic fracture	25
f	Spinal fractures	9
g	Hind legs fractures with pelvic fractures	25
h	Pelvic fracture with multiple rib fractures	18
i	Closed fracture of both fore legs with pelvic fracture	25
j	Closed fracture of both fore legs with multiple rib fractures	25

The experiments were carried out in accordance with the local guidelines for the care of laboratory animals of Zhejiang Chinese Medical University (SYXK (Zhe) 2018‐0012) and approved by the Ethics Committee for Research on Laboratory Animal Use of our institution (I ACUC‐20210111‐01).

### Gastrointestinal Function Evaluation

A semi‐solid nutrient paste was used to assess the intestinal propulsion rate in the mice. Five grams of methyl cellulose was dissolved in 125 ml of distilled water followed by the addition of 8 g of milk powder, 4 g of sugar, and 4 g of starch, with stirring after each addition. Activated carbon (1.5 g) was then added with stirring. The final volume of the nutrient paste containing activated carbon was 150 ml (150 g). The mixture was stored in a refrigerator at 4°C but warmed to room temperature before use. Before the start of the experiment, all mice were fasted for 12 h and deprived of water for 2 h. Each mouse was then given 2 g of semi‐solid nutrient paste via gavage. Eight mice in each group were then randomly selected for measurements of gastrointestinal motility.

Intestinal propulsion was assessed as follows: After the mice had been euthanized, the small intestine was removed by dissection, with the cut made at the ileocecal junction. The small intestine was then flattened on white paper without traction to measure its length. The maximum distance was that between the pyloric sphincter and end of the ileum (and thus of the small intestine). Motility was defined as the distance traveled by the active charcoal from the pyloric sphincter. Gastrointestinal motility (%) was calculated as the distance traveled by the active charcoal divided by the total length of the small intestine ×100.

### Histopathology

Histopathological changes in the intestine were examined by removing a section of jejunal tissue from the euthanized mice. The tissue was fixed in 10% neutral formaldehyde and stained with hematoxylin and eosin.

### Immunohistochemical

Jejunal tissue samples were fixed with 4% buffered paraformaldehyde for 48 h at room temperature and embedded in paraffin. Then, 4 μm sections were prepared and incubated overnight at 4°C with primary antibody against rat c‐kit diluted 1:1000 in phosphate‐buffered saline (PBS). After incubation, the sections were washed with PBS and incubated with horseradish‐peroxidase‐conjugated secondary antibody for 1 h at room temperature, and then with 2‐(4‐amidinophenyl)‐6‐indolecarbamidine dihydrochloride (DAPI) for 5 min. The sections were then observed under a light microscope (Axio Scope A1; ZEISS, Germany).

### Western Blot

Total proteins from jejunal tissue were extracted for 30 min on ice using RIPA buffer containing a proteinase inhibitor cocktail. After a 15‐min centrifugation, the supernatant was collected and the protein content was quantified using a BCA protein quantification assay. The protein samples were separated by denaturing SDS‐PAGE (8%–12%) and transferred onto a nitrocellulose membrane. The membrane was blocked with 5% non‐fat milk for 2 h at 4°C, followed by an overnight incubation at 4°C with primary antibodies against β‐actin and c‐kit (Abcam, USA). After washing, the membrane was incubated with peroxidase‐conjugated secondary antibody at 4°C for 2 h. The results were visualized using Western Lightning® Plus ECL followed by X‐ray film exposure. Each experiment was conducted in triplicate.

### Statistical Analysis

The results of the ANOVA are expressed as mean ± standard deviation. A *p*‐value <0.01 was considered to indicate a significant difference. All analyses were performed using SPSS 24.0 software (IBM Corp., USA).

## Results

### Gastrointestinal Dysfunction after Multiple Fractures

**FIGURE 1:**
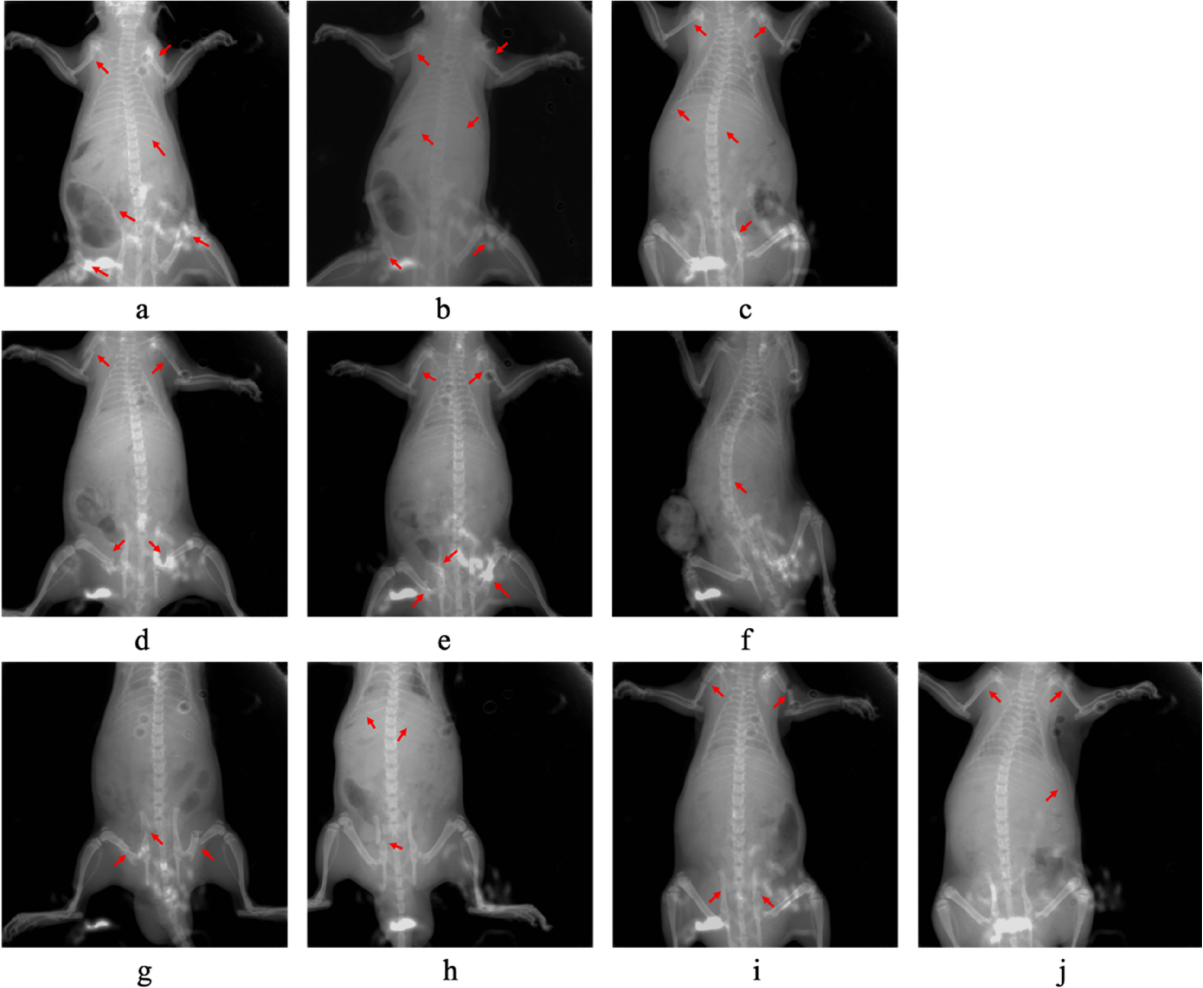
X‐ray images of the multiple fracture types described in Table 1. The fracture sites are marked with arrows

The fractures were confirmed by X‐ray (Figure 1) and accorded with the ISS, as shown in Table 1.

### Histopathological Verification of Gastrointestinal Dysfunction

**FIGURE 2:**
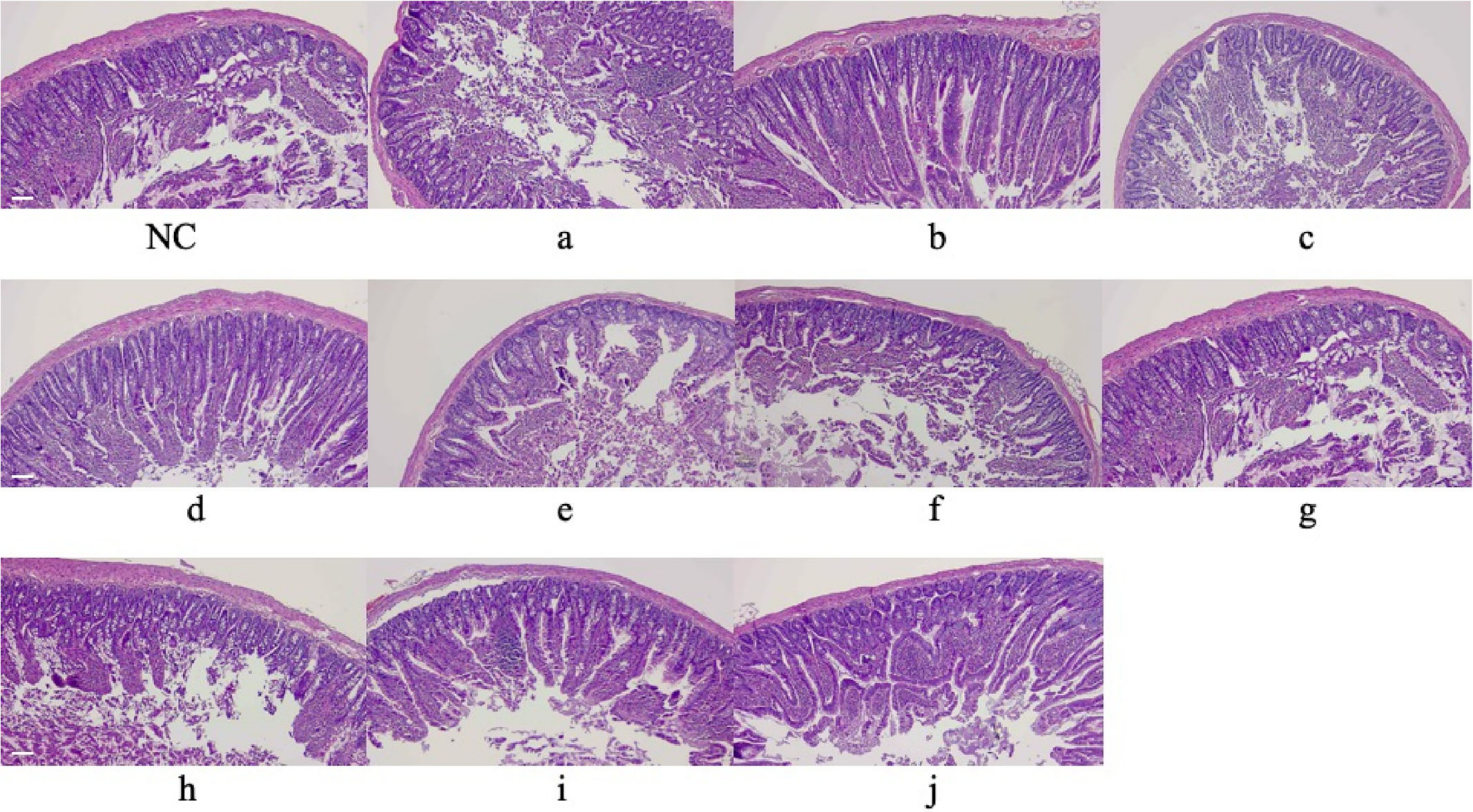
Histopathological findings in mouse jejunal tissue. NC = control. Scale bar = 100 μm

The histopathological findings were consistent with structural changes and necrosis in the small intestinal tissue of mice with multiple fractures. As shown in Figure 2, in the control group the intestinal epithelium was intact and the lamina propria glands were normal. However, in groups a, c, f, and h, there was a large amount of necrotic and exfoliated intestinal mucosal epithelial tissue and inflammatory cell infiltration in the lamina propria. Compared with groups a, c, h, g, and f, groups b, d, e, i, and j had milder intestinal epithelial necrosis and either no or mild inflammatory cell infiltration. These results suggest an association between the overall results of the histopathological observation and ISS.

### Gastrointestinal Function Evaluation

**FIGURE 3:**
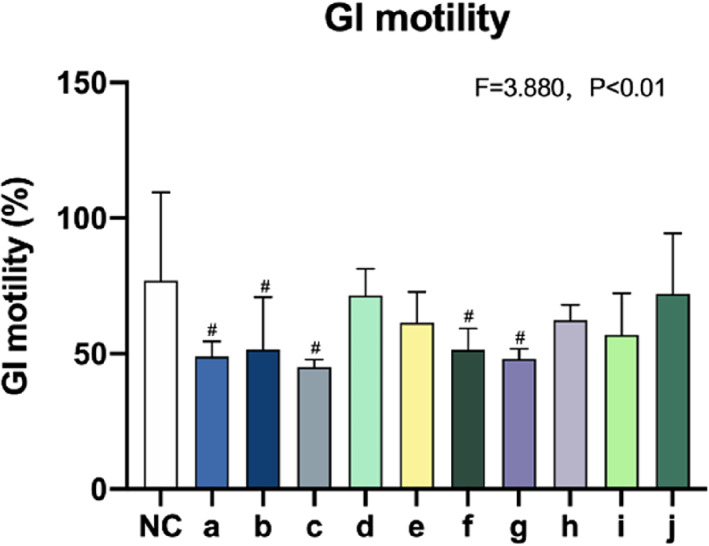
Gastrointestinal (GI) motility measured in the small intestine. Values (mean ± SD) marked with # were significantly different from the control group in least significant difference multiple comparisons

As shown in Figure 3, gastrointestinal motility was assessed as a measure of gastrointestinal function. Compared with the control group, gastrointestinal motility was decreased in groups b, c, f, and g (*p* < 0.01), i.e., those with a higher ISS, indicating that the severe damage caused by multiple fractures strongly inhibited gastrointestinal function.

### Molecular Validation of Gastrointestinal Dysfunction

**FIGURE 4:**
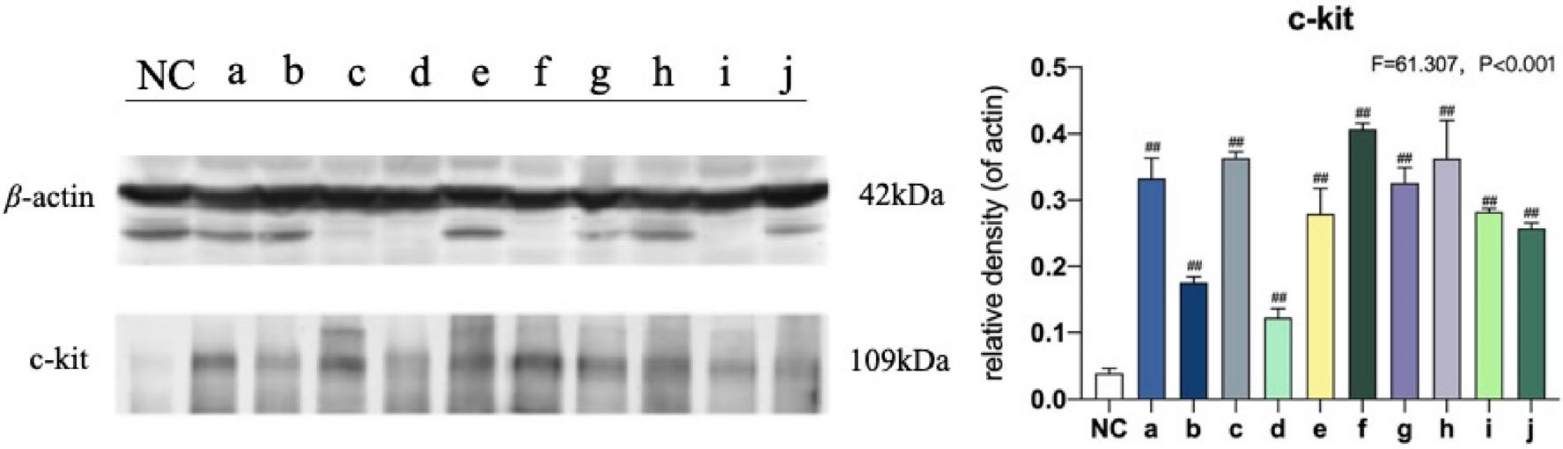
Expression and phosphorylation of c‐kit protein in mouse interstitial cells of Cajal (ICCs) in jejunal tissues. Values (mean ± SD) marked with *##* are significantly different from the control group in LSD multiple comparisons

Western blot was used to analyze c‐kit protein expression and phosphorylation in the 10 groups of mice and the control group. As shown in Figure 4, the expression of c‐kit protein was strongly up‐regulated in all model groups compared with the control group (*p* < 0.001). This result provided molecular confirmation of gastrointestinal dysfunction in mice with multiple fractures. The very high level of c‐kit protein in groups a, c, f, and h further demonstrated the close relationship between the ISS and trauma severity.

### Histopathological Verification of c‐Kit Protein Expression

**FIGURE 5:**
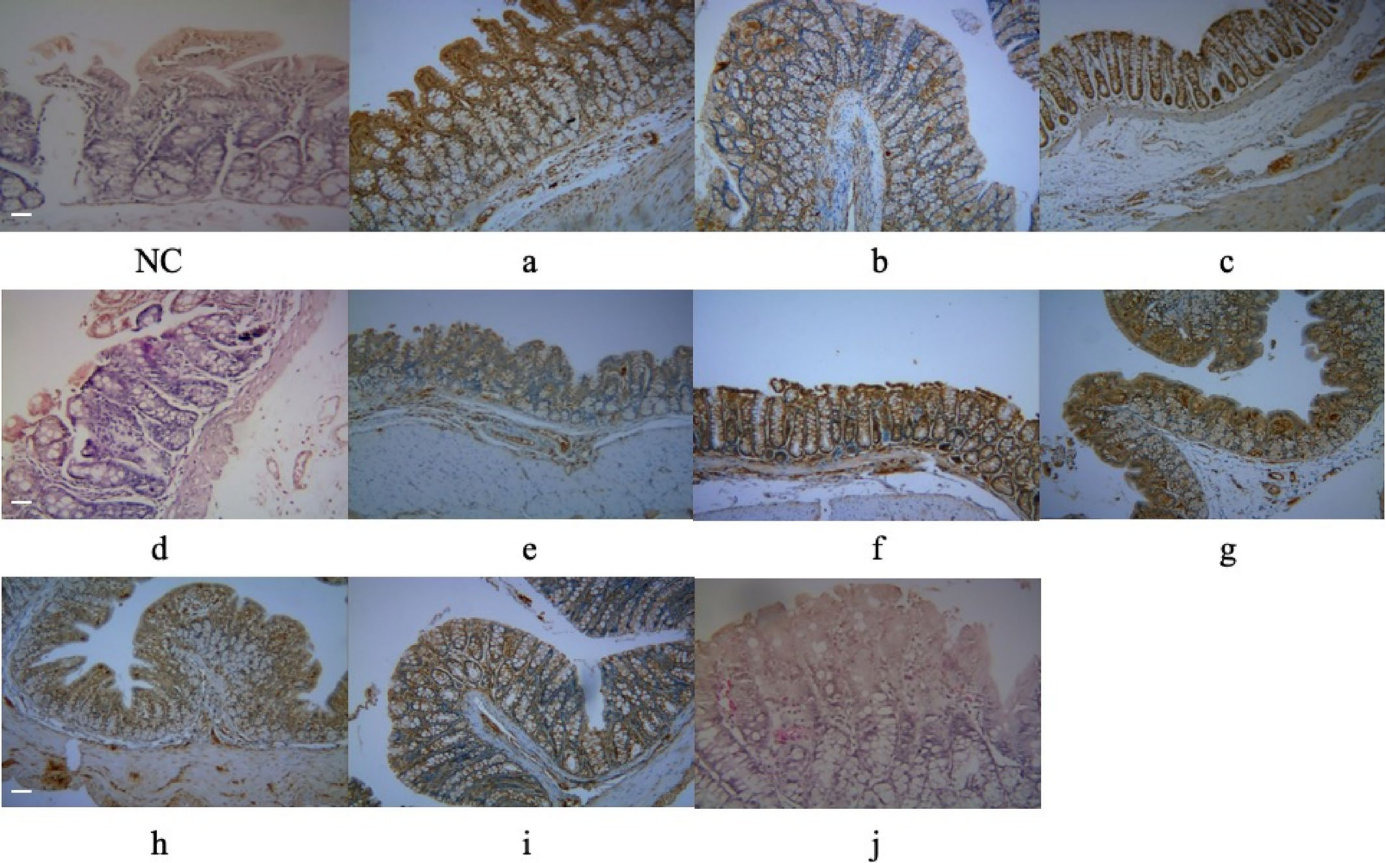
Immunohistochemical analysis of c‐kit protein in jejunal tissues. Scale bar = 100 μm

The expression of c‐kit protein in small intestinal tissue was confirmed by immunohistochemical analysis. As shown in Figure 5, expression was very strongly upregulated in the intestinal epithelium of groups a, c, f, and g compared to the control group, while in groups b, d, e, h, i, and j only slight changes were seen. These results demonstrate that c‐kit protein expression in intestinal epithelial ICCs increases with increasing severity of gastrointestinal dysfunction.

## Discussion

This study established a mouse model of gastrointestinal dysfunction developing after multiple fractures differing in severity (according to their ISS). The model was validated by analyzing c‐kit protein expression on ICCs. Applications of the model include studying c‐kit as a possible therapeutic target.

### Gastrointestinal Dysfunction Modeling

As the gastrointestinal tract is responsible for digesting food, absorbing nutrients, and releasing waste, its normal functioning is fundamental for maintaining a healthy body.^20^, ^21^ Patients with clinically relevant multiple injuries typically suffer gastrointestinal dysfunction, including a loss of appetite and indigestion, due to prolonged bed rest and limited mobility, which in turn affects their prognosis.^9^, ^10^ Currently, an animal model that accurately mimics this condition is lacking. Therefore, in this study, a mouse model of gastrointestinal dysfunction associated with multiple fractures was established. Assessments of the mice showed that multiple fractures clearly affected the motility of the small intestine, which was also evident at the histopathological level. Mice with limb, pelvic, and multiple rib fractures (group a, ISS = 34), closed fracture of both forelegs with pelvic fracture and rib fractures (group c, ISS = 34), spinal fractures (group f, ISS = 9), and hind leg and pelvic fractures (group g, ISS = 25) had severely restricted gastrointestinal motility. The pathological results for the high ISS groups indicated more severe structural damage in the jejunal tissue and a decrease in gastrointestinal motility that in some cases was >15%. Although the ISS scores were lower in mice with spinal fracture (group f, ISS = 9), the severe gastrointestinal dysfunction in this group can be explained by the fact that spinal injury causes peritoneal hematoma, which in turn strongly affects gastrointestinal function.^22^, ^23^

### Deficits in the Model

Although the experimental results demonstrated the validity of the animal model created in this study, the characteristics of the experimental animals themselves, as well as those of the injuries, must be taken into account. First, humans walk upright, while mice move using four legs. Consequently, a mouse model cannot fully simulate the impact of fracture trauma on human mobility. Furthermore, due to the anatomical differences between humans and mice, mice with closed fractures of both forelegs and pelvic fracture (group i, ISS = 25), and those with closed fracture of both forelegs and multiple rib fractures (group j, ISS = 25), were still somewhat mobile; they were able to crawl and stand for short periods of time after they were injured. In these mice, the trauma had little effect on gastrointestinal motility. Second, the influence of psychological factors in humans on gastrointestinal function cannot be reproduced in experimental animals.^24^, ^25^ Third, multiple fractures are often high‐energy injuries, which could not be simulated by our modeling method.

### Role of c‐Kit Protein and ICCs in Gastrointestinal Dysfunction

Changes in the expression of c‐kit protein in the gastrointestinal tissues of the mice were also examined. The results showed that expression was more strongly up‐regulated in mice with pelvic fractures and multiple rib fractures (group a, ISS = 34), closed fracture of both forelegs, pelvic fracture, and rib fractures (group c, ISS = 34), spinal fractures (group f, ISS = 9), and hind leg fractures with pelvic fractures (group g, ISS = 25) than in the other model groups. This result was confirmed in the immunohistochemical analysis, which showed that the expression of c‐kit protein by ICCs of the intestinal epithelium was higher in mice with severe gastrointestinal disability related to severe trauma. ICCs are involved in the regulation of slow wave propagation in the gastrointestinal tract^26^, ^27^ and the conduction of enteric nerve signals to smooth muscle,^28^, ^29^ in addition to acting as mechanical sensors.^30^ Given the important role of ICCs in gastrointestinal motility and their utility as indicators of gastrointestinal pathology, they were examined in this study. Among the experimental approaches to improving gastrointestinal motility disorders is the repair of ICC ultrastructure, as these cells have strong regenerative ability.^20^, ^31^ c‐kit protein is a member of the tyrosine growth receptor family and has multiple effects on the survival, proliferation, migration, and homing of ICCs.^32^, ^33^, ^34^, ^35^ In the colon, c‐kit promotes the proliferation and migration of intestinal epithelial cells,^36^ participates in the initiation and propagation of slow‐wave activity in gastrointestinal muscles (mediated by its receptor on ICCs).^37^, ^38^, ^39^ Our study showed that c‐kit protein of ICCs is strongly up‐regulated in response to trauma, with higher levels seen with increasing trauma severity. Elucidation of the specific mechanism in future studies may allow effective treatment of the gastrointestinal dysfunction that often develops in patients with multiple fractures.

### Strengths and Limitations

To the best of our knowledge, this is the first study to establish a mouse model of gastrointestinal dysfunction after multiple fractures differing in severity (according to their ISS). The model was used to explore the role of c‐kit protein and ICCs in gastrointestinal dysfunction. However, the limitations of the model discussed above must be taken into account. The sample size was small, only 15 in each group, which also might affect the experimental results. In addition, the surgical procedure itself may have impacted gastrointestinal function. More detailed studies of the roles of c‐kit protein and ICCs in gastrointestinal dysfunction are also needed.

### Conclusion

In this study a mouse model of gastrointestinal dysfunction after multiple fractures differing in ISS was established. An analysis of the associations among c‐kit protein, ICC, and the ISS showed that c‐kit expression reflected the severity of trauma, and thus gastrointestinal dysfunction. With the exception of characteristic spinal fractures, the degree of gastrointestinal dysfunction increased with an increase in the ISS score. Although this newly established model can simulate gastrointestinal dysfunction after multiple fractures, the fact that the characteristics of humans cannot be fully simulated in mice remains a limitation. In addition, c‐kit protein remains to be explored as a potential therapeutic target.

## Authors Contributions

MSJ: Wrote the text, collected and analyzed the data, approved the final version for submission.

FMQ: Collected and analyzed the data, approved the final version for submission.

QJS: Cases collected, collected and analyzed the data, approved the final version for submission.

ZJW: Collected and analyzed the data, approved the final version for submission.

XHH: Collected and analyzed the data, approved the final version for submission.

ZY: Collected and analyzed the data, approved the final version for submission.

ZWQ: Collected and analyzed the data, approved the final version for submission.

SLT: Designed the study, wrote the text, approved the final version for submission.

HJF: Designed the study, wrote the text, prepared the figures, approved the final version for submission.

## Conflict Of Interest Statement

There is no conflict of interest for this work.

## Ethical Approval

Experiments were carried out in accordance with the local guidelines for the care of laboratory animals of Zhejiang Chinese Medical University (SYXK (Zhe) 2018‐0012) and were approved by the ethics committee for research on laboratory animal use of the institution (I ACUC‐20210111‐01).
