# Correction to “An Innovative Model of ISS‐Based Multiple Fractures and Gastrointestinal Dysfunction Related to c‐Kit Protein Expression on Interstitial Cells of Cajal”

**DOI:** 10.1111/os.70353

**Published:** 2026-05-26

S.‐J. Meng , M.‐Q. Fan , J.‐S. Qian , J.‐W. Zhang , H.‐H. Xu , Y. Zheng , W.‐Q. Zhao , L.‐T. Shan , and J.‐F. Huang , “An Innovative Model of ISS‐Based Multiple Fractures and Gastrointestinal Dysfunction Related to c‐Kit Protein Expression on Interstitial Cells of Cajal,” Orthopaedic Surgery 15 (2023): 1325–1332, 10.1111/os.13599.36919913 PMC10157708

In Figure 2, an incorrect image of Group G was used to represent the “NC group.” The image that was published does not correspond to the correct experimental data for this group. The correct image for the NC group in Figure 2 is the representative image originally captured during the study, as shown below.

**FIGURE 2:**
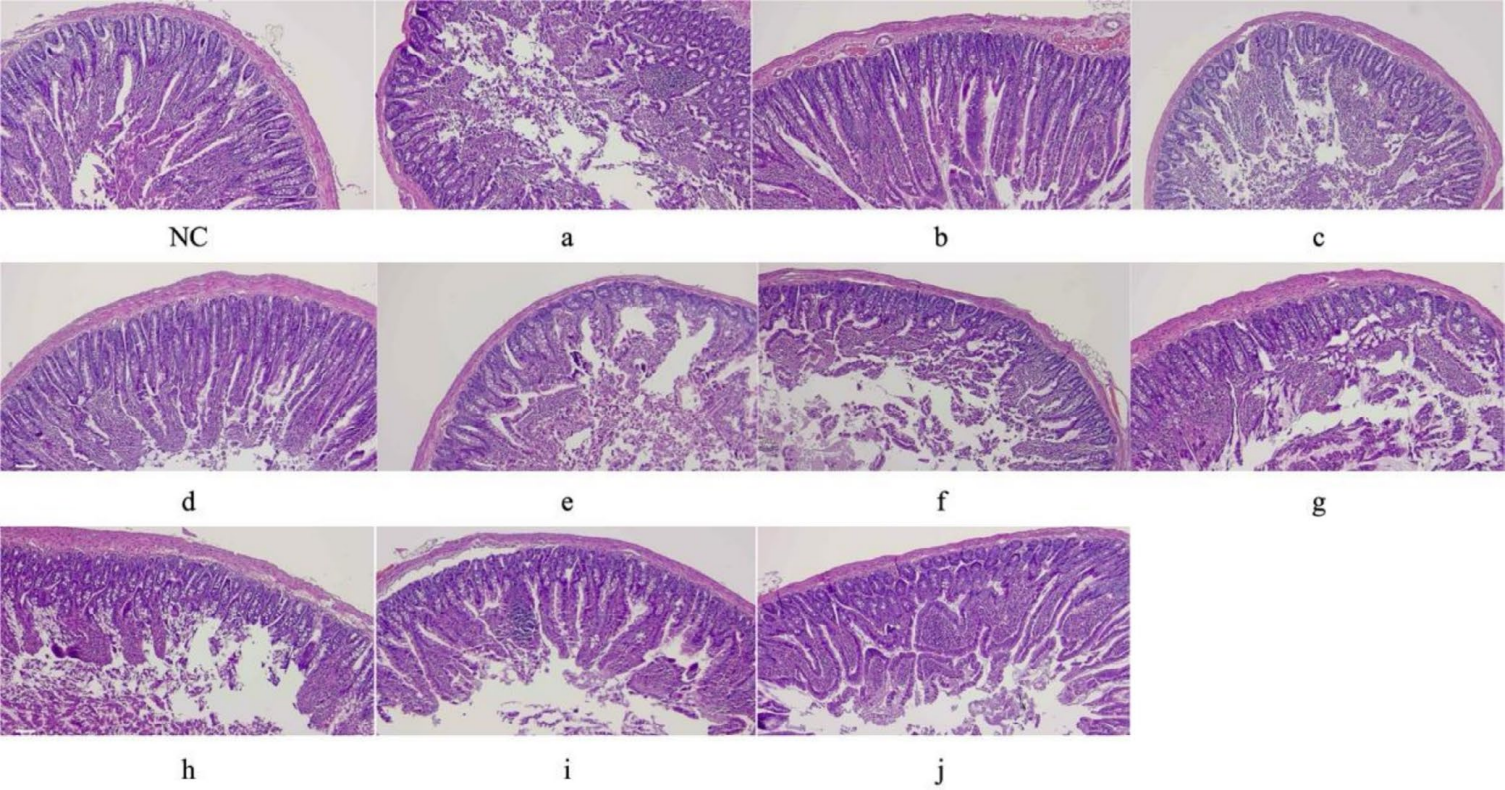
Histopathological findings in mouse jejunal tissue. NC = control. Scale bar = 100 μm.

We apologize for this error.

